# Oxycodone, an opioid like the others?

**DOI:** 10.3389/fpsyt.2023.1229439

**Published:** 2023-12-13

**Authors:** Nicolas Marie, Florence Noble

**Affiliations:** Université Paris Cité, CNRS, Inserm, Pharmacologie et Thérapies des Addictions, Paris, France

**Keywords:** oxycodone, opioids, addiction, morphine, epigenetics, gene regulations, preclinical studies

## Abstract

The over-prescription of opioid analgesics is a growing problem in the field of addiction, which has reached epidemic-like proportions in North America. Over the past decade, oxycodone has gained attention as the leading opioid responsible for the North America opioid crisis. Oxycodone is the most incriminated drug in the early years of the epidemic of opioid use disorder in USA (roughly 1999–2016). The number of preclinical articles on oxycodone is rapidly increasing. Several publications have already compared oxycodone with other opioids, focusing mainly on their analgesic properties. The aim of this review is to focus on the genomic and epigenetic regulatory features of oxycodone compared with other opioid agonists. Our aim is to initiate a discussion of perceptible differences in the pharmacological response observed with these various opioids, particularly after repeated administration in preclinical models commonly used to study drug dependence potential.

## Introduction

The over-prescription of opioid analgesics is a growing problem in the field of addiction, which has reached epidemic-like proportions in North America. Of the opioid agonists, oxycodone is the most incriminated drug in the early years of the epidemic of opioid use disorder in USA (roughly 1999–2016), but restrictions on the prescriptions and distribution of oxycodone and other synthetic opiates later resulted in its displacement by the widespread availability of non-prescribed fentanyl and its congeners. France has seen an increase in the use of prescription pain killers (including oxycodone) over the last 15 years, and health officials are concerned about the corresponding rise in addiction and overdoses, an increase that seems more marked in some regions, such as Nouvelle-Aquitaine and Bretagne.^1^

The number of preclinical articles on oxycodone is rapidly increasing. Several publications have already compared oxycodone with other opioids, focusing mainly on their analgesic properties [e.g., (1–4)]. Recently, in June 2023, Barrett et al. published a comprehensive review devoted specifically to oxycodone (5). The aim of our study is to focus on the genomic and epigenetic regulatory features of oxycodone compared with other opioid agonists. Our aim is to initiate a discussion of perceptible differences in the pharmacological response observed with these various opioids, particularly after repeated administration in preclinical models commonly used to study drug dependence potential. This area has been the subject of a number of recent publications, underlining the importance of our review.

This review aims to provide a brief historical overview of the development of oxycodone and to better understand its biological characteristics by comparing this opioid with other well-known opioids such as heroin and morphine, as well as buprenorphine for instance.

## The brief history of the development of oxycodone

The oldest trace of opium use dates back more than 7,000 years. Interestingly, it has a variety of uses, including anesthesia (6). Indeed, the milky substance obtained from incising the opium capsule contains many alkaloids that have anesthetic and analgesic properties. The primary compounds among these is morphine. It was discovered by Seguin, Courtois and Desrone and described as an alkaloid by Sertürner, between 1804 and 1817. The invention of the hypodermic syringe by Charles Gabriel Pravaz in 1850 greatly facilitated the use of morphine, particularly on various battlefields. However, it soon became apparent that morphine was addictive, just like opium. In 1874, the Bayer company synthesized heroin, a diacetylated derivative of morphine that was quickly found to be a more potent molecule than morphine. A few years later, in 1916, oxycodone was synthesized in Germany from thebaine, another alkaloid found in opium. In 1928, the Merck company introduced an injectable product called Scophedal, which included scopolamine, oxycodone and ephedrine. It caused a profound analgesia and sedation and was extensively used during the 2^nd^ world war. Its use declined after 1945 and was discontinued in 1987 (7). Meanwhile, in 1939, oxycodone appeared on the North American market, but this time not in a combination with other substances.

In the 1990s, oxycodone was often prescribed to treat pain associated with acute traumatic injuries, post-operative pain and cancer pain (8, 9). At the beginning this opioid was popular, due to its ability to improve the quality of life of patients suffering from chronic pain, with low side-effects compared to morphine. However, after few years of prescription there is a lack of evidence regarding the long-term effectiveness of oxycodone, and it clearly appears that long-term oxycodone therapy involves serious health and dependency risks. The production of an extended-release form of oxycodone did not lead to a reduce abuse liability of the drug [review of the driving factors that contribute to misuse of oxycodone in Kibaly et al. (10)].

Thus, at this time oxycodone is considered the most commonly misused prescription opioid, but a change in physician prescription practices in 2009 made prescription opioids less accessible, especially for first-time opioid users (11). Paradoxically total opioid-related overdose deaths between 2013 and 2017 increased substantially, from 25,052 to 47,600 in USA, which was primarily driven by fentanyl and its synthetic, mostly illicit analogs (12). In many cases, fentanyl and its analogs are added to heroin, cocaine and amphetamine-like stimulants, and all these combinations are fueling the opioid epidemic in the U.S. and worldwide (13).

## Binding properties

Historically, three opioid receptors have been characterized: mu (MOR), delta (DOR) and kappa (KOR), which have been cloned in the beginning of the 90’s. Morphine is the prototypal opioid with a high affinity for MOR (in the nM range) and good selectivity, with approximately 50 times less affinity for KOR and no binding to DOR (14).

Oxycodone (6-deoxy-7,8-dihydro-14-hydroxy-3-O-methyl-6-oxomorphine) is a semi-synthetic opioid synthesized from thebaine. It has a lower affinity for MOR compared to morphine (5 to 40 times lower, depending on the studies) and binds to KOR and DOR with very low affinity, in the μM range (Table 1). Oxycodone is mainly metabolized into noroxycodone by CYP3A4/5 and into oxymorphone via CYP2D6. These two metabolites are ultimately transformed into noroxymorphone. While noroxycodone is inactive, oxymorphone and noroxymorphone are active metabolites. Oxymorphone has a higher affinity than oxycodone (16) for MOR and also a greater efficacy and potency (measured by receptor coupling) (4). It is used as an analgesic in both veterinary and human medicine. Noroxymorphone, such as oxymorphone, has greater efficacy and potency toward MOR (26).

**Table 1:** Pharmacokinetic and pharmacodynamic properties of opioid ligands and their metabolites.

	Metabolites	Binding parameters	Transporters on BBB	Ref and (*species*)
Heroin		low affinity to MOR (Ki 158 nM) MOR>DOR (Ki 3,895 nM) > KOR (Ki 5,634 nM)	No substrate of P-gp	(15) (*mouse*)
				(16) (*rat*)
Heroin	6-monoacetylmorphine (6-MAM)		No substrate of P-gp	(15) *(mouse)*
	Morphine	MOR (Ki 3.35 nM) > KOR (Ki 96.4 nM) > DOR (Ki 195 nM)	P-gp substrate	(17) (*human*)
			MRP substrate	(15) *(mouse)*
Oxycodone		MOR (Ki 43.9 nM) > DOR (Ki 2,160 nM) > KOR (Ki 5,943 nM) Partial mu agonist	P-gp substrate	(16) *(rat)*
Oxycodone	Nor-oxycodone	MOR (Ki 57 nM) >> > KOR and DOR (KI > 1,000 nM)	Lower uptake into the rat brain compared to oxycodone	(18) *(human)*
	Nor-oxymorphone	MOR (Ki 5 nM) > KOR (Ki 80 nM) > DOR (160 nM)		(18) *(human)*
	Oxymorphone	MOR (Ki 0.98 nM) > KOR (Ki 80 nM) > DOR (Ki 84.20 nM) Partial mu agonist	lower uptake into the rat brain compared to oxycodone	(18) (*human*)
				(17) *(human)*
			MRP substrate	(16) *(rat)*
Buprenorphine		Partial MOR agonist	P-gp substrate	(19)
		DOR antagonist		(20)
		KOR antagonist		(21)
		ORL1 agonist		(22) (*rat MOR, mouse DOR, human KOR and ORL1*)
		MOR (Ki 0.08 nM) > KOR (Ki 0.11 nM) > DOR (Ki 0.42 nM) > ORL1 (Ki 285 nM)		
Buprenorphine	Norbuprenorphine	MOR (Ki 0.07 nM) > KOR (Ki 0.91 nM) > DOR (Ki 0.91 nM) >> > ORL1 (Ki 7,330 nM)	P-gp substrate	(23) *(mouse)*
				(22)
	Buprenorphine-3-glucuronide	MOR (Ki 4.9 pM) > DOR (Ki 270 nM) > ORL1 (36 μM). No affinity for KOR		(24) (*human*)
	Norbuprenorphine-3-glucuronide	ORL1 (Ki 18 μM) > MOR (Ki 0.3 μM). No affinity for DOR and KOR		(24) *(human)*
Morphine		MOR (Ki 1.7 nM) > KOR (Ki 65.5 nM) > DOR (KI 104.6 nM) Partial mu agonist	P-gp substrate	(17) *(human)*
				(15) *(mouse)*
			MRP substrate	(16) *(rat)*
Morphine	Morphine-3-glucuronide	MOR (Ki 40 nM)	MRP substrate	(21)
	Morphine-6-glucuronide	MOR (Ki 0.6 nM)	MRP substrate	(25) *(rat)*
Hydromorphone		MOR (Ki 0.5 nM) > DOR (Ki 9.08 nM) > KOR (12.9 nM)		(16) *(rat)*

Interestingly, heroin is a weak MOR ligand with about 100 times lower affinity than morphine (16). It behaves as a prodrug; indeed, it is rapidly converted to 3-(3-MAM) and 6-Monoacetylmorphine (6-MAM, the most active metabolite) and finally to morphine. Regarding their activity on MOR, all three opiates (heroin, morphine, oxycodone) activate MOR with similar efficacy but with differences in potency, as measured in the GTPγS assay: morphine > oxycodone > > heroin (16). Efficacy (Emax) is the capacity of a drug to produce a maximum response, and potency is the amount of drug needed to produce a certain amount of response.

Regarding other opioid ligands, the binding affinity to MOR of hydromorphone is around 3 times greater than that of morphine (27). Buprenorphine is an oripavine derivative with mixed agonist–antagonist activity at classical opioid receptors, mu, delta, kappa and ORL-1. In this way, buprenorphine is a unique drug with a complex pharmacology [see (19)]. Buprenorphine is a potent partial MOR agonist with a very high affinity (0.08 nM), and with a long duration of action related to a very slow receptor kinetics/receptor dissociation rates (28, 29). In pioneering studies conducted in rodents, buprenorphine displayed a ceiling effect, exerting only partial analgesia compared to morphine or more effective agonists (30). Nevertheless, more recent studies have not shown this ceiling effect in other species such as humans where buprenorphine is quite powerful (31).

## Blood–brain barrier, and rate of delivery to the brain

Despite their common core structure, heroin, oxycodone and morphine have different pharmacokinetic properties. Owing to the presence of two acetyl groups, heroin is the most lipophilic, with a miLogP (calculated with https://www.molinspiration.com) of 1.61 compared to morphine and oxycodone, which have miLogP values of 1.1 and 0.79, respectively. Therefore, heroin rapidly reaches the brain after intravenous injection, with a Tmax of 1.5 min in rat brain extracellular fluid (32). Once in the brain, it is sequentially hydrolyzed into 6-Monoacetylmorphine (6-MAM) and then into morphine (33). Both of these metabolites activate MOR. In fact, 6-MAM like morphine (see above) is a potent MOR agonist (34). Regarding morphine, its lower lipophilicity, combined with its uptake by efflux pump such as Pgp (P-glycoprotein), slows its brain penetration. The Tmax in brain cortical microdialysate after subcutaneous injection is 45 min (35). Interestingly, the partition coefficient for hydromorphone is almost twice that of morphine, which explains why hydromorphone is approximately 6–8 times more potent than morphine whereas the binding affinity reported is only 3 times greater (27). Oxycodone penetrates the blood brain barrier (BBB) well, likely with the help of active transport (36), resulting in a fast onset of action. Therefore, the onset of analgesic effect is observed very rapidly after intravenous administration in humans (Tmax ~6 min) (37, 38) and after 15 min in rats after intraperitoneal or subcutaneous administration (39). In both cases, it is faster than morphine. Metabolites of oxycodone, noroxymorphone and oxymorphone have a reduced ability to penetrate the blood–brain barrier (40, 41).

## Molecular adaptations following chronic treatments

### Gene regulations

The consequences of chronic opioid treatment are manifested by increased drug craving, tolerance development and expression of withdrawal symptoms when the opioid is discontinued. Each of these features is a result of adaptive changes in the expression levels of several genes. Numerous genes have been identified in different brain regions that are either upregulated or downregulated in responses to repeated opioid exposure. However, comparing published results has always been a challenge because opioid exposure and subsequent drug withdrawal induce different phase-specific temporal gene expressions. Thus, different gene regulations may be observed during drug exposure, shortly after termination of drug-exposure, or after long-period of abstinence [e.g., (42); see Table 2].

**Table 2:** Gene regulations in rodents following different opioid treatments.

Opioid used	Models	Mode of treatment	Methodologies to investigate gene expression	Criterion to genes selection	Brain structures	Specificity	Main regulations observed	References
Morphine	Male Wistar rats	Daily morphine injections (10 mg/kg morphine i.p.) for 14 days, and withdrawal for 3 weeks.	RT-qPCR. Tissue collection at different time point: during exposure and abstinence. 159 genes analyzed	Fold change cut-off ≥1.35	Nucleus accumbens	Morphine exposure phase	Time-dependent regulation: dopamine receptors (*D1*, *D2L*, *D2S*, *D3*), MOR, melanocortin receptor (*MC4-R*), substance P receptor (*subP-R*), *5HT1b-R*, receptors *CB1, 5HT1d*, muscarinic acetylcholine receptors (*mACh-R m1*, *mACh-R m2*), kainate and NMDA subunits of the glutamate receptor type (*Glu5-7*, *NR1*, *NR2A*, *NR2B*)	(42)
						Expression profiles in the abstinence phase	*Downregulated: EphrinA4, #11.4, Arc, MENI, junD, NAC, NARF, GABA α2, rGβ, nAChR α2, GluR2, Zfp40, Gephyrin, GABA β3, GSK3β, GAP-43, 5HT3R, Synaptotag, TGFR, Neurodap, PLA2, EphrinB2, GR*	
							*Upregulated: GABA β2, subPR, SORLA, trkA, nAChR β4, GluR4, #10.1, NR2a, μ-OR, EphRA7, #27, krox-20, EGFR, synaptoph, GluR3, Gβ3, IP3R, GABA ε, GABA β1, sub P, Neureguline*	
Morphine	Male wistar rats	Animals received for 10 days twice daily ascending doses of morphine injections intraperitoneally according to the following schedule: days 1 and 2: 2 × 10 mg/kg; days 3 and 4: 2 × 20 mg/kg, days 5 and 6: 2 × 30 mg/kg, days 7 and 8: 2 × 40 mg/kg, days 9 and 10: 2 × 50 mg/kg. On the morning of day 11, animals received a morphine (50 mg/kg), and 2 h later the last dose of morphine (50 mg/kg).	Microarray Hybridization	Fold change cut-off ≥ 2	Frontal cortex	Brain were removed 2 h after the last dose of morphine	Using DNA microarray analysis 14 out of 8,000 genes were induced at least twofold in the frontal cortex of rats chronically treated with morphine as compared to saline treated control animals: *Heat shock protein 70 (hsp70), Heat shock protein 27 (hsp27), EST197399 (unknown), rPer2, Prolyl 4-hydroxylase a subunit, Myosin alkali light chain, exon 6, hsp40, a-crystallin B chain, ania-3, arc, hsp 105, GRP78, p23 (p58/p45), Immunoglobulin heavy chain binding protein (BiP)*	(43)
							Only one gene was reduced more than twofold: *3-Hydroxy-3-methylglutaryl coenzyme A reductase*.	
Morphine	Male and female Sprague Dawley rats	Daily injections of s.c. morphine (5.0 mg/kg) over 10 consecutive days.	RNA-seq and RT-qPCR	For RNA-seq: z-score ≥ 2; value of *p* <0.05	Prefrontal coretx	Brain collection 24 h after the 10th injection	In male rats, 377 genes were differentially expressed in the morphine-treated relative to the saline-treated group, among which 337 (89%) were upregulated and 40 (11%) were downregulate.	(44)
							In female rats, 409 genes were significantly differentially expressed in morphine-treated relative to saline-treated rats, with 370 (90%) upregulated and 39 (10%) downregulated.	
							Male and female groups shared a subset of 204 (35%) differentially expressed genes: coding for neurotransmitter receptor subunits (*Chrna7, Chrnb2, Gabra4, Gabrb2, Gabrb3, Grin3a, Grm5, Htr2a, Htr5a, Pgr*), intra−/inter-cellular signaling regulation (*Camk2d, Cdk5r1, Efnb2, Kalrn, Prkaa2, Prkacb, Prkce*), and synaptic morphology/function (*Stx1b, Syngr1, Synpo, Syt1*)	
Oxycodone	Male Sprague–Dawley rats	15 mg/kg i.p. every 12 h for 8 days	Microarray Hybridization	Fold change ≥ 1.5, *p* < 0.05	Whole-brain tissues (including olfactory tubercules) were harvested, the cerebellums were excluded.	12 h after administration of the last dose.	Upregulated genes in brain tissues of oxycodone-treated rats: *PAIHC3 (ITIH3), Dusp6 (MKP-3), Sgk1, Gpd1a, RGD1311086, Fkbp5a predicted, Abcg2, Gpd1, Sult1a1, Rpe65, Fkbp5 predicted, Mt1a (metallothionein-1X), RGD1311086b predicted, Per2, RGD1311086 predicted, Smpdl3b predicted, Dio2, Lims2 predicted (PINCH-2), Klf15 (KLF15), Cryab (Alpha crystalline B), Thrsp (S14 protein), Dsipi DSIPI (GILZ), Net1 predicted (SRX1), Nt5 (5 -NTD), Usp2 (UBP41), RGD1309044 predicted, Tac2 (Nk3)*	(45)
							Downregulated genes in brain tissues of oxycodone-treated rats: *Slc16a1 (MCT1), Nkx2-2 predicted, Cklfsf6 predicted, LOC498276 (Fc γ RII α), Col4a1 predicted, RGD1307925 predicted (TBC1D23), Akap1, Erbb3, Tmem27 (Collectrin), Serpinh1 (HSP47), Dpyd, Gna12 (G-protein alpha-12), Rffl, Prss35, Scap2, Rt1.Da (HLADRA), Map1b, Kdr (VEGFR-2), Tnfrsf11b (Osteoprotegrin), Tgm2 (STAM2), Adamts1 (ADAM-TS1), Tm4sf1 predicted [TM4SF1 (TAAl6)], S100a8 (Calgranulin A), S100a9 (Calgranulin B)*	
Oxycodone	Male Holtzman rats	Rats were trained to respond under a fixed-ratio (FR) 1 schedule in which each press of an active lever produced an infusion of 0.06 mg/kg i.v. After 10 sessions under the FR1 schedule, the FR value was increased to FR2 and FR3 for five sessions each. Sessions were 120 min in duration and ran 5 days per week.	RT-qPCR	*p* < 0.05	Dorsal striatum	Brain collection 24 h after the last oxycodone self-administration session	Downregulation: *Adcy5*	(46)
							Upregulation: *Egr2*	
					Nucleus accumbens		Upregulation: *c-fos, Egr2*	
Oxycodone	Male C57BL/6 J mice	A 4-h self-administration session was carried out every day for 14 consecutive days. A nose poke at the active hole led to an infusion of oxycodone (0.25 mg/kg/infusion) under a FR1 schedule.	RNA-seq	value of *p* < 0.05	Nucleus accumbens	Brain collection 1 h after the last self-administration session	Upregulation: Opioid system: *Pomc, Oprd1*; Stress system: *Fkbp5, Cry1*; Neurotransmitter systems: *Ankk1, Htr1b, Htr2a, Tph2, Drd2*; Kinases and Transcription factors: *Nr1h2, Pim1, Epha4, Htra1, Arc, Gsk3b*	(47)
							Downregulation: Opioid system: *Oprl1, Pnoc*; Stress system: *Crhr2*; Neurotransmitter systems: *Htr7, Glra1, Galr1, Gabrb2, Gabra1, Grin3a, Cdh23, Gabrg1, Npy2r, Chrnb2, Gabrg2, Chrm5, Gad1, Chrm2*	
Heroin	Male Sprague–Dawley rats	I.v. self-administration under FR10 ratio. 6 h self-administration session, 5 days a week for a total of 16 trials.	RNA-seq	fold change cut-off ≥ 1.25	Prefrontal cortex	24 h after a extinction/reinstatment session, and 48 h after the last i.v.self-administration trial.	*Saline vs heroin escalating group Upregulated: Arhgef28, Slc38a9, Pde7a, Ttc22, Fxyd6, Pcdhga5, RGD1561507, Dcx.*	(48)
							*Downregulated: Zfp865, Lsp1, Bche, Gbp2, Rbms1, Zfp775, Skap2, Pigv, Dhrs3, Esyt1, Atf7, Casp1, Sema3f, Rnf182, Rpp40, Psmb9, Tap1, Ccdc167, Adora2a*	

To obtain a global profile of genes regulated by repeated oxycodone administration (8 days 15 mg/kg i.p., twice daily, with sacrifice 12 h after the last injection) Affymetrix microarrays have been used. Oxycodone regulates numerous genes that are involved in important biological processes, including drug metabolism, immune response, organic anion transport, antigen presentation via MHC class II molecule, the dopamine receptor signaling pathway, and the transmembrane receptor tyrosine kinase signaling pathway. In this study, the authors observed that many genes regulated by oxycodone have previously been reported to be modulated by morphine as well (43, 45).

Several genes that have previously been shown to be regulated by chronic opiate treatment also appear regulated following oxycodone exposure. This was observed 24 h after the last oxycodone self-administration session in rats (2 h/day, 5 day/week, 20 sessions) in the dorsal striatum and/or nucleus accumbens. Oxycodone induces the downregulation of *Adcy5* mRNA in the dorsal striatum, upregulation of c-Fos in the nucleus accumbens, and upregulation of Egr2 in both structures (46). In mice, 1 h after the last oxycodone administration, in a model of intravenous self-administration (4 h/day, 14 consecutive days), significant regulation of 5 genes was observed in the ventral striatum, *Htr7*, *Glra1*, *Galr1*, *Htr2a* and *Pomc* (47).

Globally, all opioid agonists are able to regulate genes of opioid system coding for MOR, KOR and DOR (49–53) and for endogenous opioid peptides (proopiomelanocortin, prodynorphin, proenkephalin) (54–56), plasticity (*Arc*, *Bdnf*, *Npy*, *Cdh2*…), stress (*Avpr1a*, *Crh*, *Crhr1*, *Crhr2*, *Nr3c1*, *Fkbp5*), and kinases and signaling [*Akt1*, *Arrb1*, *Arrb2*, *Mapk1*…; e.g., (57)]. However, differences in the activation levels of signaling molecules may be observed among opioid (58). These differences could be attributed to variations in experimental procedures and/or on more complex factors. In a recent study, distinct transcriptional responses to oxycodone and buprenorphine were reported in induced pluripotent stem cells-derived brain organoids from patients with opioid use disorder. Oxycodone primarily affected transcriptional responses in neurons, whereas buprenorphine significantly regulated transcription in glial cells. Specifically, oxycodone, but not buprenorphine, was found to induce STAT1, a transcription factor that interacts with several genes in the interferon signaling pathway (59). The pharmacokinetics and /or pharmacodynamics properties of the different opioids may also be responsible of differences in gene and protein regulations. For instance, repeated treatment with oxycodone was shown to increase the expression of Psd95 in the hippocampus (60), similar results were observed with heroin in the nucleus accumbens (61), but a decrease was observed in both the prefrontal cortex and the hippocampus following treatment with morphine (62). The reasons for these regulatory differences are unknown. However, as mentioned earlier, it is well established that oxycodone, morphine, and heroin exhibit distinct pharmacokinetic (PK) and pharmacodynamic (PD) characteristics.

### Activation of ERK pathway

Previous studies have demonstrated that addictive drugs can increase the phosphorylation of ERK in specific brain regions. Furthermore, the activation of ERK induces the phosphorylation of various transcription factors, including CREB, which regulates genes and protein expression involved in addictive processes. Table 3 reports homogeneous results regarding ERK and CREB phosphorylation in a conditioned place preference model, regardless of opioid use or animal model. In mice, both fentanyl and morphine increase the phosphorylation of ERK and CREB in the hippocampus, nucleus accumbens, and prefrontal cortex, but not in the striatum (63–65). In rats, some differences may be observed between morphine and oxycodone in certain brain structures, such as the hippocampus and prefrontal cortex, concerning both phosphoERK and phosphoCREB (66–68). As a transcription factor, CREB could regulate many targets (69) and some could be retrieved among the gene regulated by opioids (Table 2). For instance, genes coding for Neuregulin, GABA subunits or NMDA subunits are CREB targets (70–72), and were found to be upregulated after opioid treatment (see Table 2). Similarly, activation of the ERK pathway initiates cell-specific gene regulation necessary for changes in synaptic efficacy. And both CREB and ERK pathways have been shown to regulate circadian clock genes (73–75), which are regulated by morphine and oxycodone [Table 2; (43, 45)].

**Table 3:** Regulation of phospho-ERK and phospho-CREB by opioid-induced conditioned pace preference.

Opioid-induced CPP	Animals	Treatment	phosphoCREB	phosphoERK	References
Fentanyl	Male C57BL/6 mice	0.05 mg/kg i.p.	↑Hippocampus	↑Hippocampus	(63)
			↑Accumbens	↑Accumbens	
			↑Prefrontal cortex	↑Prefrontal cortex	
			= Striatum	= Striatum	
Morphine	Male C57BL/6 mice	10 mg/kg i.p.	↑Hippocampus	↑Hippocampus	(64)
			↑Accumbens	↑Accumbens	
Morphine	Male C57BL/6 mice	10 mg/kg i.p.	↑Hippocampus	↑Hippocampus	(65)
Morphine	Male Wistar rats	5 mg/kg s.c.	↑Accumbens	↑Accumbens	(66)
			↑Amygdala	↑Amygdala	
			↑Striatum	↑Striatum	
			↑Prefrontal cortex	↑Prefrontal cortex	
Morphine	Male Wistar rats	10 mg/kg i.p.	= Hippocampus	↑Hippocampus	(67)
			= Cerebral cortex	↑Cerebral cortex	
Oxycodone	Wistar rats	2.5 mg/kg s.c.	↑Accumbens	↑Accumbens	(68)
			↑Hippocampus	↑Hippocampus	
			= Prefrontal cortex	= Prefrontal cortex	

### Epigenetics regulation

Gene regulation may involve epigenetic changes that include post-translational histone modifications (mainly methylation and acetylation), DNA methylation, and miRNAs. Thus, opioids have been reported to induce histone modifications (76, 77), such as increased histone H3 acetylation, which plays a crucial role in heroin- or morphine-induced conditioned place preference (CPP), suggesting the involvement of this post-translational modification in opioid-mediated behaviors. This hypothesis is supported by the evidence showing that inhibition of histone deacetylases (HDAC) enhances morphine-associated memory formation (78) and promotes the reinstatement of heroin-seeking induced by heroin priming in heroin intravenous self-administration paradigms (79). Moreover, these changes in histone acetylation observed in rodents are consistent with a study reporting increased acetylation of H3K27 in the post-mortem striatum of heroin addicts (80). Interestingly, this histone modification can regulate the expression of glutamate receptor subunits, in accordance with the data reported in Table 2. Publications specifically investigating histone modifications by oxycodone are relatively scarce. One paper reported that an inhibitor of bromodomain and extra-terminal (BET) proteins, a class of histone acetylation readers, was unable to alter oxycodone-induced CPP in mice (81). In another study, using a model of intravenous self-administration, it was shown that oxycodone leads to an increase in histone H3 phosphorylation at serine 10 and acetylation at lysine 14 (82). Interestingly, this histone acetylation is mediated by CREB pathway, which is consistent with other studies that have shown that oxycodone activates CREB (Table 3).

Changes in histone and DNA methylation have also been reported after opioid exposure (76, 77). Thus, it was shown that oxycodone induces a decrease in global DNA methylation, with modifications in the expression of genes involved in synaptic function and plasticity (e.g.*, arc* that is regulated by opioids, see Table 2), as well as regulation in the transcription of DNA methyltransferases (DNMTs) (60, 83). DNMTs are responsible for adding methyl groups to cytosine-guanine dinucleotides (CpGs) in the genome. In mammals, the main DNMTs include DNMT1 which is responsible for DNA methylation maintenance and another two “*de novo*” methyltransferases that establish new methylation patterns (DNMT3A and DNMT3B). It has been observed that opioids may regulate theses DNMT, although the specific effects may vary depending on the opioid and the experimental models. Thus, a decrease in the expression of *DNMT1* was observed in the hippocampus in all phases of oxycodone-induced CPP (acquisition, expression, extinction and reinstatement) (60), while an increase of DNMT1 in the nucleus accumbens was observed in a model of heroin self-administration (84). DNMT3 expression was increased in the hippocampus of rats exposed to morphine self-administration (85), but not in the CPP with oxycodone (60), or in heroin self-administration (84).

Gene expression can also be regulated at the transcription and translation levels by non-coding RNA, including miRNA and long noncoding RNA. However, only a few studies have been performed with opioids. In a clinical study, comparing acute administration of hydromorphone and oxycodone in healthy subjects, of 179 plasma miRNAs measured, 9 miRNAs were commonly upregulated and 17 miRNAs were commonly downregulated (86). The authors attributed these results to the different pharmacodynamic properties of both opioids, with hydromorphone primarily binding to MOR and to a lesser extent to DOR, while oxycodone activates multiple receptors, including KOR. In another study, overexpression of miR-9 was reported in serum of metamphetamine but not heroin abusers (87). MiR-9 is of interest as it has been found to play a critical role in drug addiction-associated hippocampal synaptic plasticity and memory by directly affecting the expression of genes related to impaired hippocampal long-term potentiation (88). MiR-9 also has been shown to directly or indirectly regulate a number of genes involved in reward function, including dopamine D2 receptors (89), which is regulated by chronic morphine and oxycodone treatments as shown in Table 2 (42, 47). A preclinical study demonstrated that chronic morphine treatment decreased the expression of miR-9 in the prefrontal cortex (90). However, this regulation appears to be complex and dependent on the type of drug and the brain region analyzed, as cocaine was shown to increase miR-9 expression in the nucleus accumbens, and decrease its expression in the dorsal striatum (91). Regulation in the nucleus accumbens is certainly crucial in addiction, as overexpression of miR-9 in this brain region increases escalation of oxycodone self-administration (92).

In conclusion, it is well established that opioids induce numerous transcriptional and epigenetic regulations. However, differences may also arise depending on the specific opioid agonist used, although comparisons may be challenging due to differences in exposure and protocols across studies. These differences may also be influenced by pharmacokinetic and pharmacodynamic properties (93), including factors such as affinity, intrinsic efficacy, and speed of crossing the blood–brain barrier.

## Behavioral consequences of chronic opioid treatments

Addiction is a complex brain disease that affects behavior in various ways. It is characterized by compulsive drug-seeking and drug-taking behaviors, as well as loss of control over drug intake, despite negative effects on health, social interactions, and occupational functions. Repeated exposure to opioids leads to long-lasting neuroadaptations that contribute to the behavioral changes associated with addiction. Rodents provide an accurate model for studying these addictive behaviors.

### Locomotor sensitization

Sensitization is a phenomenon in which a specific behavioral, physiological, or cellular response increases over time following repeated exposure to a particular drug of abuse. These sensitized responses are long-lasting and can persist for weeks or months. One commonly studied measure in preclinical studies is the sensitization of locomotor activity induced by drugs of abuse. Behavioral sensitization paradigms are believed to mimic the lasting maladaptive changes in the brain that occur after repeated drug intake, leading to increased sensitivity to the neurobiological effects of abused drugs. These adaptations are believe to contribute to an increased propensity for intake, abuse, and relapse. All opioid agonists have the ability to induce locomotor sensitization, but some differences may be observed. In previous studies (94, 95) we highlighted the importance of the specific agonist used to promote sensitization, as well as the patterns of drug administration. In these studies mice were treated with escalating doses of morphine, methadone or buprenorphine during 5 days given either once (binge) or three times a day (TTD). Methadone and morphine were found to induce locomotor sensitization under both conditions (binge and TTD), whereas locomotor sensitization was restricted to binge treatment with buprenorphine. Moreover the sensitization observed with buprenorphine was less than that observed with methadone. These differences could be explained by the partial mu opioid agonistic properties of buprenorphine, as compared to full agonists like methadone or morphine. Moreover buprenorphine also has a long half-life [about 3 h (96)] and a slow dissociation rate from mu opioid receptor (29).

On the other hand, while locomotor sensitization is observed following repeated treatments with morphine, heroin, and oxycodone (94, 97, 98), it is interesting to see that this effect is stronger in adolescent rodents compared to adults with oxycodone (99) and morphine (100), but not with heroin (101). These results point out that the effects of opioid agonists often differ. The reasons for these differences are not yet known, but they may involve variations in pharmacokinetic and pharmacodynamic properties, as previously reported regarding the molecular adaptations following chronic treatments.

### Conditioned place preference

Another popular paradigm utilized in modeling opiate addiction is the conditioned place preference approach. A typical CPP experiment is conducted using a two-compartment apparatus that incorporates unique environmental cues (i.e., tactile, visual) in each compartment, or a three-compartment apparatus with a neutral (non-paired) chamber. During conditioning, the animal receives repeated passive drug injections followed by confinement to one of the two compartments, allowing the formation of associations between drug effects and contextual cues, and the other compartment with a neutral substance such as saline. Following conditioning, animals are tested in a drug-free state, and if the animal spends more time in the drug-conditioned compartment, then the drug is considered to have a rewarding effect.

The CPP paradigm can also be used to model relapse. After standard conditioning and testing for a place preference, animals undergo extinction either by additional conditioning with only saline in both compartments, or by repeated placement into the testing apparatus, which reduces preference for the drug-paired compartment in the absence of additional drug conditioning sessions. Following extinction, animals are exposed to either stress or a priming injection of the original conditioning drug, which are sufficient to reinstate CPP, and thus serve as potential models of relapse (102, 103).

All opioid agonists, including the partial agonist buprenorphine, are able to induce a CPP, and no differences in the magnitude of morphine-, oxycodone-, and heroin-induced CPP have been observed (104). One limitation of the CPP paradigm is the lack of a clear dose dependency, although some studies have described opioid agonist-induced CPP as dose-dependent (105). However, it has been suggested that the persistence of CPP extinction may be an alternative measure to assess the rewarding efficacy of drug doses in cases of non-dose-dependent effects. Interestingly, it appears that more extinction sessions are required to extinguish morphine-induced CPP compared to oxycodone and heroin (104).

Another intriguing finding from comparing different opioid agonists in CPP is that a nociceptin agonist reduces oxycodone-induced CPP (with a right-shift of the minimal active dose by 100-fold), while a smaller effect was observed with morphine (2-fold shift) or heroin (3-fold shift) (106). Since the rewarding effects of opioids are mediated by increased dopaminergic activity in the mesocorticolimbic system, and nociceptin receptors in the brain can reduce dopamine levels (107–112), it is plausible to speculate that the observed differences in CPP reflect varying impacts of the opioid agonist on dopamine release. This is consistent with the study of Vander Weele et al. (113) which demonstrated dramatic differences between morphine and oxycodone in their patterns of drug-evoked dopamine transmission. Oxycodone induced a long lasting dopamine release in the nucleus accumbens lasting more than 35 min, while morphine produced a brief increase in dopamine levels, significant only during the first minute following drug administration. The implications of dopamine levels quickly returning to baseline after morphine delivery, but not after oxycodone administration are not yet clear, but this difference may contribute to the high misuse of oxycodone and the opioid crisis.

### Intravenous self-administration paradigms

The intravenous self-administration model is one of the paradigms used in preclinical addiction studies. Prior to 2017, the number of publications on self-administration models with oxycodone was very small. However, there has been a significant increase in publications since then, although still relatively small compared to the number of publications on psychostimulants. Currently, publications on oxycodone represent about 20% of the publications on opioids (source: PubMed, using keywords: oxycodone or opioid/self-administration/rat). Several models have been developed using intravenous self-administration. By modifying the daily duration of access to the drug, two distinct populations of animals can be obtained. Animals with short access per day (e.g., 1 h per day, referred to as ShA for Short Access) typically exhibit controlled and limited drug use during self-administration session. On the other hand, animals with prolonged access (e.g., 6 h per day, referred to as LgA for Long Access), tend to show a rapid escalation of drug intake that becomes excessive and compulsive. Therefore, LgA rats exhibit behavioral characteristics that are indicative of addiction, and this model has been characterized and validated with multiple substances of abuse (e.g., cocaine, heroin, methamphetamine) (114–117). However, some discrepancies can be observed, particularly with oxycodone. While some authors have reported a lack of escalation during restricted access (1 or 3 h) (118, 119), a recent study has highlighted that the number of infusions and active lever presses increased over the course of the sessions under both LgA and ShA conditions (120).

In a recent study (121) we compared the effects of morphine, heroin and oxycodone using the LgA model of intravenous administration. In this experiment, self-administration trials were conducted 5 days per week, and a total of 22 sessions were performed. All three opioids resulted in the development a self-administration behavior in the rats. However some differences could be observed. One of the notable difference was that while heroin consumption showed a gradual increase, the patterns were different for morphine and oxycodone. With these two opioids, we consistently observed peaks in consumption during the sessions conducted after 2 days without access to the drug. The reasons for these differences between heroin, on one hand, and morphine and oxycodone, on the other hand, are difficult to explain. One hypothesis could be related to the pharmacokinetic properties of the drugs used. Heroin has a very rapid neural effect following intravenous administration compared to morphine and oxycodone (see “Blood–brain barrier, rate of delivery to the brain” section). Therefore, it can be speculated that when the craving is strong after 2 days without the drug, the animal may exhibit a behavior aimed at achieving a faster increase in brain concentrations. This behavior may involve a higher number of injections in an attempt to rapidly raise the cerebral concentrations, especially with molecules that have a low Tmax (such as morphine and oxycodone). This behavior is likely unnecessary with heroin because it reaches the brain very quickly, providing rapid relief.

Another intriguing finding observed (121) was the heterogeneity within the LgA groups exposed to heroin and oxycodone, in contrast to the morphine LgA group. When calculating the ratio between the number of presses on the active lever and the number of injections, a clear distinction emerged. The morphine-exposed rats exhibited a highly homogeneous pattern, as the ratio was close to 1 for all animals (indicating an equal number of lever presses and infusions). However, in the case of heroin and oxycodone, the animals could be divided into two distinct groups: those with a ratio close to 1, and those with a ratio > 1.5 (indicating a higher number of lever presses than infusions, likely due to lever presses during the 20-s timeout following an infusion). This specific pattern may reflect compulsive behavior. Approximately one-third of the rats exhibited such behavior with both heroin and oxycodone. This observation is in line with recent studies showing that in rats with long access to drugs, two different populations can be distinguished: those who maintain moderate, controlled drug consumption, and those who show an escalation in their consumption (119, 122).

Based on these findings, it could be suggested that oxycodone and heroin have a stronger potential for abuse than morphine, and some rats may develop behavior that can be described as compulsive. Although all three opioids studied may have abuse potential, as previously observed in the clinic (123), when considering some behavioral parameters measured in the intravenous self-administration paradigm, morphine may be considered safer, while oxycodone and heroin exhibit more risky behaviors such as escalation of use and the development of compulsive behavior in some individuals. However, it is important to note that craving is likely significant for all these opioids, including morphine, as suggested with a peak of injections systematically observed after 2 days without access to the drug.

Several human studies have also examined the dependence potential of oxycodone compared with other opioids. This review focuses mainly on preclinical studies, however, and will not go into detail. Nevertheless, it is worth mentioning that in opioid-dependent subjects, oxycodone is generally identified as the more desirable drug compared with substances such as methadone or morphine (124). This may be explain by the favorable pharmacokinetic parameters of oxycodone, e.g., better brain penetration, formation of long half-life metabolites, longer dopamine release [review in Kibaly et al. (10)].

## Conclusion

The aim of this review was to provide a brief overview, with a focus on dependence, of the preclinical data obtained with oxycodone and to compare the results with those obtained with other opioid ligands, especially morphine and heroin. These two agonists are widely used, with morphine known for its analgesic properties and heroin used for drug abuse. This analysis clearly shows that, while these three opioids share a common target, MOR, they lead to different molecular and cellular regulations, and thus to different behavioral adaptations. It is not yet clear how we can explain these differences, however one of the key features may be differences in pharmacodynamic and pharmacokinetic properties, as described here. These differences make each opioid ligand unique.

Opioids are known to induce numerous side-effects, among them overdoses have become a serious health issue, especially in case of overuse. Most opioid-related deaths are caused by respiratory depression, which essentially involve activation of mu opioid receptors. Interestingly, as reported in the present review in the field of dependence, some differences may be observed between morphine, heroin, and oxycodone regarding brain oxygen changes following administration of these opioid ligands. Thus, Kiyatkin (125) reports that morphine and oxycodone were clearly less potent to induce brain hypoxia than heroin, and these differences could be due to specific pharmacokinetic properties.

## Author contributions

All authors listed have made a substantial, direct, and intellectual contribution to the work and approved it for publication.

## Footnotes

^1^https://ansm.sante.fr/actualites/antalgiques-opioides-lansm-publie-un-etat-des-lieux-de-la-consommation-en-france

## References

[1] Dinges H-C Schubert A-K Rücker G Otto S Waldmann S Wiesmann T. Equianalgesic potency ratios of opioids used in patient-controlled analgesia: a network meta-analysis. J Opioid Manag. (2022) 18:567–86. doi: 10.5055/jom.2022.0751, PMID: 36523208

[2] Ing Lorenzini K Daali Y Dayer P Desmeules J. Pharmacokinetic-pharmacodynamic modelling of opioids in healthy human volunteers. A minireview. Basic Clin Pharmacol Toxicol. (2012) 110:219–26. doi: 10.1111/j.1742-7843.2011.00814.x, PMID: 21995512

[3] Kalso E Pöyhiä R Onnela P Linko K Tigerstedt I Tammisto T. Intravenous morphine and oxycodone for pain after abdominal surgery. Acta Anaesthesiol Scand. (1991) 35:642–6. doi: 10.1111/j.1399-6576.1991.tb03364.x 1785245

[4] Kalso E. Oxycodone. J Pain Symptom Manag. (2005) 29:47–56. doi: 10.1016/j.jpainsymman.2005.01.010 15907646

[5] Barrett JE Shekarabi A Inan S. Oxycodone: a current perspective on its pharmacology, abuse and Pharmacotherapeutic developments. Pharmacol Rev. (2023):PHARMREV-AR-2021-000506. doi: 10.1124/pharmrev.121.000506, PMID: 37321860 PMC10595024

[6] Merlin MD. Archaeological evidence for the tradition of psychoactive plant use in the Old World. Econ Bot. (2003) 57:295–323. doi: 10.1663/0013-0001(2003)057[0295:AEFTTO]2.0.CO;2

[7] Defalque RJ Wright AJ. Scophedal (SEE) was it a fad or a miracle drug? Bull Anesth Hist. (2003) 21:12–4. doi: 10.1016/s1522-8649(03)50051-8 17494237

[8] Schmidt-Hansen M Bennett MI Arnold S Bromham N Hilgart JS. Efficacy, tolerability and acceptability of oxycodone for cancer-related pain in adults: an updated Cochrane systematic review. BMJ Support Palliat Care. (2018) 8:117–28. doi: 10.1136/bmjspcare-2017-001457, PMID: 29331953

[9] Schmidt-Hansen M Bennett MI Arnold S Bromham N Hilgart JS Page AJ. Oxycodone for cancer-related pain. Cochrane Database Syst Rev. (2022) 2022:CD003870. doi: 10.1002/14651858.CD003870.pub7, PMID: 25723351

[10] Kibaly C Alderete JA Liu SH Nasef HS Law P-Y Evans CJ. Oxycodone in the opioid epidemic: high “liking”, “wanting”, and abuse liability. Cell Mol Neurobiol. (2021) 41:899–926. doi: 10.1007/s10571-020-01013-y, PMID: 33245509 PMC8155122

[11] Cicero TJ Ellis MS Kasper ZA. Increased use of heroin as an initiating opioid of abuse. Addict Behav. (2017) 74:63–6. doi: 10.1016/j.addbeh.2017.05.030 28582659

[12] Imtiaz S Shield KD Fischer B Elton-Marshall T Sornpaisarn B Probst C. Recent changes in trends of opioid overdose deaths in North America. Subst Abuse Treat Prev Policy. (2020) 15:66. doi: 10.1186/s13011-020-00308-z, PMID: 32867799 PMC7457770

[13] Jones CM Bekheet F Park JN Alexander GC. The evolving overdose epidemic: synthetic opioids and rising stimulant-related harms. Epidemiol Rev. (2020) 42:154–66. doi: 10.1093/epirev/mxaa011, PMID: 33511987 PMC9200066

[14] Raynor K Kong H Chen Y Yasuda K Yu L Bell GI. Pharmacological characterization of the cloned kappa-, delta-, and mu-opioid receptors. Mol Pharmacol. (1994) 45:330–4. PMID: 8114680

[15] Seleman M Chapy H Cisternino S Courtin C Smirnova M Schlatter J. Impact of P-glycoprotein at the blood-brain barrier on the uptake of heroin and its main metabolites: behavioral effects and consequences on the transcriptional responses and reinforcing properties. Psychopharmacology. (2014) 231:3139–49. doi: 10.1007/s00213-014-3490-9, PMID: 24705903

[16] Peckham EM Traynor JR. Comparison of the antinociceptive response to morphine and morphine-like compounds in male and female Sprague-Dawley rats. J Pharmacol Exp Ther. (2006) 316:1195–201. doi: 10.1124/jpet.105.094276, PMID: 16291875

[17] Dumitrascuta M Bermudez M Ben Haddou T Guerrieri E Schläfer L Ritsch A. N-Phenethyl substitution in 14-Methoxy-N-methylmorphinan-6-ones turns selective μ opioid receptor ligands into dual μ/δ opioid receptor agonists. Sci Rep. (2020) 10:5653. doi: 10.1038/s41598-020-62530-w, PMID: 32221355 PMC7101422

[18] Lalovic B Kharasch E Hoffer C Risler L Liu-Chen L-Y Shen DD. Pharmacokinetics and pharmacodynamics of oral oxycodone in healthy human subjects: role of circulating active metabolites. Clin Pharmacol Ther. (2006) 79:461–79. doi: 10.1016/j.clpt.2006.01.009 16678548

[19] Lutfy K Cowan A. Buprenorphine: a unique drug with complex pharmacology. Curr Neuropharmacol. (2004) 2:395–402. doi: 10.2174/1570159043359477, PMID: 18997874 PMC2581407

[20] Suzuki T Zaima C Moriki Y Fukami T Tomono K. P-glycoprotein mediates brain-to-blood efflux transport of buprenorphine across the blood-brain barrier. J Drug Target. (2007) 15:67–74. doi: 10.1080/10611860601141606, PMID: 17365275

[21] Chaves C Remiao F Cisternino S Decleves X. Opioids and the blood-brain barrier: a dynamic interaction with consequences on drug disposition in brain. Curr Neuropharmacol. (2017) 15:1156–73. doi: 10.2174/1570159X15666170504095823, PMID: 28474563 PMC5725546

[22] Huang P Kehner GB Cowan A Liu-Chen LY. Comparison of pharmacological activities of buprenorphine and norbuprenorphine: norbuprenorphine is a potent opioid agonist. J Pharmacol Exp Ther. (2001) 297:688–95., PMID: 11303059

[23] Liao MZ Gao C Shireman LM Phillips B Risler LJ Neradugomma NK. P-gp/ABCB1 exerts differential impacts on brain and fetal exposure to norbuprenorphine. Pharmacol Res. (2017) 119:61–71. doi: 10.1016/j.phrs.2017.01.018, PMID: 28111265 PMC5392442

[24] Brown SM Holtzman M Kim T Kharasch ED. Buprenorphine metabolites, buprenorphine-3-glucuronide and norbuprenorphine-3-glucuronide, are biologically active. Anesthesiology. (2011) 115:1251–60. doi: 10.1097/ALN.0b013e318238fea0, PMID: 22037640 PMC3560935

[25] Chen ZR Irvine RJ Somogyi AA Bochner F. Mu receptor binding of some commonly used opioids and their metabolites. Life Sci. (1991) 48:2165–71. doi: 10.1016/0024-3205(91)90150-a, PMID: 1851921

[26] Lemberg KK Heiskanen TE Kontinen VK Kalso EA. Pharmacology of oxycodone: does it explain why oxycodone has become a bestselling strong opioid?. Scandinavian Journal of Pain. (2009) 1:S18–S23. doi: 10.1016/S1877-8860(09)70005-9

[27] Volpe DA McMahon Tobin GA Mellon RD Katki AG Parker RJ Colatsky T. Uniform assessment and ranking of opioid μ receptor binding constants for selected opioid drugs. Regul Toxicol Pharmacol (2011) 59:385–390. doi: 10.1016/j.yrtph.2010.12.007 21215785

[28] Yassen A Olofsen E Dahan A Danhof M. Pharmacokinetic-pharmacodynamic modeling of the antinociceptive effect of buprenorphine and fentanyl in rats: role of receptor equilibration kinetics. J Pharmacol Exp Ther. (2005) 313:1136–49. doi: 10.1124/jpet.104.082560 15701707

[29] Mégarbane B Marie N Pirnay S Borron SW Gueye PN Risède P. Buprenorphine is protective against the depressive effects of norbuprenorphine on ventilation. Toxicol Appl Pharmacol. (2006) 212:256–67. doi: 10.1016/j.taap.2005.08.002, PMID: 16169027

[30] Cowan A. Buprenorphine: the basic pharmacology revisited. J Addict Med. (2007) 1:68–72. doi: 10.1097/ADM.0b013e31806c9202, PMID: 21768937

[31] Dahan A Yassen A Romberg R Sarton E Teppema L Olofsen E. Buprenorphine induces ceiling in respiratory depression but not in analgesia. Br J Anaesth. (2006) 96:627–32. doi: 10.1093/bja/ael051, PMID: 16547090

[32] Gottås A Øiestad EL Boix F Vindenes V Ripel Å Thaulow CH. Levels of heroin and its metabolites in blood and brain extracellular fluid after i.v. heroin administration to freely moving rats. Br J Pharmacol. (2013) 170:546–56. doi: 10.1111/bph.12305, PMID: 23865556 PMC3791993

[33] Dinis-Oliveira RJ. Metabolism and metabolomics of opiates: a long way of forensic implications to unravel. J Forensic Legal Med. (2019) 61:128–40. doi: 10.1016/j.jflm.2018.12.005, PMID: 30621882

[34] Selley DE Cao CC Sexton T Schwegel JA Martin TJ Childers SR. Mu opioid receptor-mediated G-protein activation by heroin metabolites: evidence for greater efficacy of 6-monoacetylmorphine compared with morphine. Biochem Pharmacol. (2001) 62:447–55. doi: 10.1016/S0006-2952(01)00689-X, PMID: 11448454

[35] Barjavel M Sandouk P Plotkine M Scherrmann JM. Morphine and morphine metabolite kinetics in the rat brain as assessed by transcortical microdialysis. Life Sci. (1994) 55:1301–8. doi: 10.1016/0024-3205(94)90069-8, PMID: 7934632

[36] Okura T Hattori A Takano Y Sato T Hammarlund-Udenaes M Terasaki T. Involvement of the pyrilamine transporter, a putative organic cation transporter, in blood-brain barrier transport of oxycodone. Drug Metab Dispos. (2008) 36:2005–13. doi: 10.1124/dmd.108.022087 18606742

[37] Leow KP Cramond T Smith MT. Pharmacokinetics and pharmacodynamics of oxycodone when given intravenously and rectally to adult patients with cancer pain. Anesth Analg. (1995) 80:296–302. doi: 10.1097/00000539-199502000-00016, PMID: 7818116

[38] Kokki M Välitalo P Kuusisto M Ranta VP Raatikainen K Hautajärvi H. Central nervous system penetration of oxycodone after intravenous and epidural administration. Br J Anaesth. (2014) 112:133–40. doi: 10.1093/bja/aet337, PMID: 24131664

[39] Pöyhiä R Kalso EA. Antinociceptive effects and central nervous system depression caused by oxycodone and morphine in rats. Pharmacol Toxicol. (1992) 70:125–30. doi: 10.1111/j.1600-0773.1992.tb00441.x 1508838

[40] Galetta S Hahn EF Nishimura S Pasternak GW. Oxymorphone-naltrexonazine, a mixed opiate agonist-antagonist. Life Sci. (1987) 41:783–7. doi: 10.1016/0024-3205(87)90459-0, PMID: 2441221

[41] Lemberg KK Siiskonen AO Kontinen VK Yli-Kauhaluoma JT Kalso EA. Pharmacological characterization of noroxymorphone as a new opioid for spinal analgesia. Anesth Analg. (2008) 106:463–70. doi: 10.1213/ane.0b013e3181605a15 18227301

[42] Spijker S Houtzager SWJ De Gunst MCM De Boer WPH Schoffelmeer ANM Smit AB. Morphine exposure and abstinence define specific stages of gene expression in the rat nucleus accumbens. FASEB J. (2004) 18:848–50. doi: 10.1096/fj.03-0612fje, PMID: 15033927

[43] Ammon S Mayer P Riechert U Tischmeyer H Höllt V. Microarray analysis of genes expressed in the frontal cortex of rats chronically treated with morphine and after naloxone precipitated withdrawal. Brain Res Mol Brain Res. (2003) 112:113–25. doi: 10.1016/s0169-328x(03)00057-3, PMID: 12670709

[44] Liu SX Gades MS Swain Y Ramakrishnan A Harris AC Tran PV. Repeated morphine exposure activates synaptogenesis and other neuroplasticity-related gene networks in the dorsomedial prefrontal cortex of male and female rats. Drug Alcohol Depend. (2021) 221:108598. doi: 10.1016/j.drugalcdep.2021.108598 33626484 PMC8026706

[45] Hassan HE Myers AL Lee IJ Chen H Coop A Eddington ND. Regulation of gene expression in brain tissues of rats repeatedly treated by the highly abused opioid agonist, oxycodone: microarray profiling and gene mapping analysis. Drug Metab Dispos. (2010) 38:157–67. doi: 10.1124/dmd.109.029199, PMID: 19786507 PMC2802418

[46] Pravetoni M Pentel PR Potter DN Chartoff EH Tally L LeSage MG. Effects of an oxycodone conjugate vaccine on oxycodone self-administration and oxycodone-induced brain gene expression in rats. PLoS One. (2014) 9:e101807. doi: 10.1371/journal.pone.0101807 25025380 PMC4099132

[47] Zhang Y Liang Y Randesi M Yuferov V Zhao C Kreek MJ. Chronic oxycodone self-administration altered reward-related genes in the ventral and dorsal striatum of C57BL/6J mice: an RNA-seq analysis. Neuroscience. (2018) 393:333–49. doi: 10.1016/j.neuroscience.2018.07.032, PMID: 30059705

[48] McFalls AJ Imperio CG Woodward E Krikorian C Stoltsfus B Wronowski B. An RNA-seq study of the mPFC of rats with different addiction phenotypes. Brain Res Bull. (2022) 191:107–20. doi: 10.1016/j.brainresbull.2022.09.023, PMID: 36223840

[49] Li X Sun L He J Chen Z Zhou F Liu X. The kappa-opioid receptor is upregulated in the spinal cord and locus ceruleus but downregulated in the dorsal root ganglia of morphine tolerant rats. Brain Res. (2010) 1326:30–9. doi: 10.1016/j.brainres.2010.02.070 20206145

[50] Prenus RV Luscar E Zhu Z-P Badisa RB Goodman CB. Regulation of mammalian MOR-1 gene expression after chronic treatment with morphine. Int J Mol Med. (2012) 30:1493–7. doi: 10.3892/ijmm.2012.1132, PMID: 22992838 PMC3789025

[51] Suzuki S Chuang TK Chuang LF Doi RH Chuang RY. Morphine upregulates kappa-opioid receptors of human lymphocytes. Adv Exp Med Biol. (2001) 493:81–7. doi: 10.1007/0-306-47611-8_10, PMID: 11727785

[52] Gupta A Gullapalli S Pan H Ramos-Ortolaza DL Hayward MD Low MJ. Regulation of opioid receptors by their endogenous opioid peptides. Cell Mol Neurobiol. (2021) 41:1103–18. doi: 10.1007/s10571-020-01015-w, PMID: 33389463 PMC8277103

[53] Cabañero D Célérier E García-Nogales P Mata M Roques BP Maldonado R. The pro-nociceptive effects of remifentanil or surgical injury in mice are associated with a decrease in delta-opioid receptor mRNA levels: prevention of the nociceptive response by on-site delivery of enkephalins. Pain. (2009) 141:88–96. doi: 10.1016/j.pain.2008.10.011, PMID: 19058913

[54] Chadzinska M Starowicz K Scislowska-Czarnecka A Bilecki W Pierzchala-Koziec K Przewlocki R. Morphine-induced changes in the activity of proopiomelanocortin and prodynorphin systems in zymosan-induced peritonitis in mice. Immunol Lett. (2005) 101:185–92. doi: 10.1016/j.imlet.2005.05.009, PMID: 15979727

[55] Gonzalez-Nunez V Jimenez González A Barreto-Valer K Rodríguez RE. In vivo regulation of the μ opioid receptor: role of the endogenous opioid agents. Mol Med. (2013) 19:7–17. doi: 10.2119/molmed.2012.00318, PMID: 23348513 PMC3592933

[56] Nikoshkov A Drakenberg K Wang X Horvath MC Keller E Hurd YL. Opioid neuropeptide genotypes in relation to heroin abuse: dopamine tone contributes to reversed mesolimbic proenkephalin expression. Proc Natl Acad Sci U S A. (2008) 105:786–91. doi: 10.1073/pnas.0710902105, PMID: 18184800 PMC2206614

[57] Randesi M Contoreggi NH Zhou Y Rubin BR Bellamy JR Yu F. Sex differences in neuroplasticity- and stress-related gene expression and protein levels in the rat Hippocampus following oxycodone conditioned place preference. Neuroscience. (2019) 410:274–92. doi: 10.1016/j.neuroscience.2019.04.047, PMID: 31071414 PMC7746310

[58] Emery MA Bates MLS Wellman PJ Eitan S. Differential effects of oxycodone, hydrocodone, and morphine on activation levels of signaling molecules. Pain Med. (2016) 17:908–14. doi: 10.1111/pme.12918, PMID: 26349634

[59] Ho M-F Zhang C Moon I Zhu X Coombes BJ Biernacka J. Single cell transcriptomics reveals distinct transcriptional responses to oxycodone and buprenorphine by iPSC-derived brain organoids from patients with opioid use disorder. Mol Psychiatry. (2022). doi: 10.1038/s41380-022-01837-8, PMID: 36302966 PMC10588459

[60] Fan X-Y Shi G Zhao P. Reversal of oxycodone conditioned place preference by oxytocin: promoting global DNA methylation in the hippocampus. Neuropharmacology. (2019) 160:107778. doi: 10.1016/j.neuropharm.2019.107778, PMID: 31526808

[61] Xia B Li Y Li R Yin D Chen X Li J. Effect of Sirtuin-1 on synaptic plasticity in nucleus Accumbens in a rat model of heroin addiction. Med Sci Monit. (2018) 24:3789–803. doi: 10.12659/MSM.910550, PMID: 29870523 PMC6016439

[62] Guo H Xie Q Cui J Xu D Deji C Chen Y. Naloxone reversed cognitive impairments induced by repeated morphine under heavy perceptual load in the 5-choice serial reaction time task. J Neurosci Res. (2019) 97:1051–65. doi: 10.1002/jnr.24427, PMID: 31081159

[63] Du K Wang Z Zhang H Zhang Y Su H Wei Z. Levo-tetrahydropalmatine attenuates the acquisition of fentanyl-induced conditioned place preference and the changes in ERK and CREB phosphorylation expression in mice. Neurosci Lett. (2021) 756:135984. doi: 10.1016/j.neulet.2021.135984, PMID: 34029649

[64] Zhang J Wang N Chen B Wang Y He J Cai X. Blockade of cannabinoid CB1 receptor attenuates the acquisition of morphine-induced conditioned place preference along with a downregulation of ERK, CREB phosphorylation, and BDNF expression in the nucleus accumbens and hippocampus. Neurosci Lett. (2016) 630:70–6. doi: 10.1016/j.neulet.2016.07.047 27461790

[65] Liu L Zhu J Zhou L Wan L. RACK1 promotes maintenance of morphine-associated memory via activation of an ERK-CREB dependent pathway in hippocampus. Sci Rep. (2016) 6:20183. doi: 10.1038/srep20183, PMID: 26830449 PMC4735742

[66] Haghparast A Fatahi Z Alamdary SZ Reisi Z Khodagholi F. Changes in the levels of p-ERK, p-CREB, and c-fos in rat mesocorticolimbic dopaminergic system after morphine-induced conditioned place preference: the role of acute and subchronic stress. Cell Mol Neurobiol. (2014) 34:277–88. doi: 10.1007/s10571-013-0011-z 24292370 PMC11488949

[67] Khezri A Mohsenzadeh MS Mirzayan E Bagherpasand N Fathi M Abnous K. Quetiapine attenuates the acquisition of morphine-induced conditioned place preference and reduces ERK phosphorylation in the hippocampus and cerebral cortex. Am J Drug Alcohol Abuse. (2022) 48:422–32. doi: 10.1080/00952990.2022.2069574, PMID: 35658689

[68] Liu Y-L Yan L-D Zhou P-L Wu C-F Gong Z-H. Levo-tetrahydropalmatine attenuates oxycodone-induced conditioned place preference in rats. Eur J Pharmacol. (2009) 602:321–7. doi: 10.1016/j.ejphar.2008.11.031 19071107

[69] Zhang X Odom DT Koo S-H Conkright MD Canettieri G Best J. Genome-wide analysis of cAMP-response element binding protein occupancy, phosphorylation, and target gene activation in human tissues. Proc Natl Acad Sci U S A. (2005) 102:4459–64. doi: 10.1073/pnas.0501076102, PMID: 15753290 PMC555478

[70] Grabenstatter HL Russek SJ Brooks-Kayal AR. Molecular pathways controlling inhibitory receptor expression. Epilepsia. (2012) 53:71–8. doi: 10.1111/epi.12036, PMID: 23216580 PMC3776022

[71] Lau GC Saha S Faris R Russek SJ. Upregulation of NMDAR1 subunit gene expression in cortical neurons via a PKA-dependent pathway. J Neurochem. (2004) 88:564–75. doi: 10.1046/j.1471-4159.2003.02156.x 14720206

[72] Turner JR Ray R Lee B Everett L Xiang J Jepson C. Evidence from mouse and man for a role of neuregulin 3 in nicotine dependence. Mol Psychiatry. (2014) 19:801–10. doi: 10.1038/mp.2013.104, PMID: 23999525 PMC3877725

[73] Koyanagi S Hamdan AM Horiguchi M Kusunose N Okamoto A Matsunaga N. cAMP-response element (CRE)-mediated transcription by activating transcription factor-4 (ATF4) is essential for circadian expression of the Period2 gene. J Biol Chem. (2011) 286:32416–23. doi: 10.1074/jbc.M111.258970, PMID: 21768648 PMC3173173

[74] Goldsmith CS Bell-Pedersen D. Diverse roles for MAPK signaling in circadian clocks. Adv Genet. (2013) 84:1–39. doi: 10.1016/B978-0-12-407703-4.00001-3, PMID: 24262095 PMC4437509

[75] Pačesová D Volfová B Červená K Hejnová L Novotný J Bendová Z. Acute morphine affects the rat circadian clock via rhythms of phosphorylated ERK1/2 and GSK3β kinases and Per1 expression in the rat suprachiasmatic nucleus. Br J Pharmacol. (2015) 172:3638–49. doi: 10.1111/bph.13152, PMID: 25828914 PMC4507165

[76] Browne CJ Godino A Salery M Nestler EJ. Epigenetic mechanisms of opioid addiction. Biol Psychiatry. (2020) 87:22–33. doi: 10.1016/j.biopsych.2019.06.027, PMID: 31477236 PMC6898774

[77] Reid KZ Lemezis BM Hou T-C Chen R. Epigenetic modulation of opioid receptors by drugs of abuse. Int J Mol Sci. (2022) 23:11804. doi: 10.3390/ijms231911804 36233105 PMC9569510

[78] Wang Y Lai J Cui H Zhu Y Zhao B Wang W. Inhibition of histone deacetylase in the basolateral amygdala facilitates morphine context-associated memory formation in rats. J Mol Neurosci. (2015) 55:269–78. doi: 10.1007/s12031-014-0317-4 24829091

[79] Chen W-S Xu W-J Zhu H-Q Gao L Lai M-J Zhang F-Q. Effects of histone deacetylase inhibitor sodium butyrate on heroin seeking behavior in the nucleus accumbens in rats. Brain Res. (2016) 1652:151–7. doi: 10.1016/j.brainres.2016.10.007, PMID: 27742468

[80] Egervari G Landry J Callens J Fullard JF Roussos P Keller E. Striatal H3K27 acetylation linked to glutamatergic gene dysregulation in human heroin abusers holds promise as therapeutic target. Biol Psychiatry. (2017) 81:585–94. doi: 10.1016/j.biopsych.2016.09.015, PMID: 27863698 PMC5346335

[81] Babigian CJ Wiedner HJ Wahlestedt C Sartor GC. JQ1 attenuates psychostimulant- but not opioid-induced conditioned place preference. Behav Brain Res. (2022) 418:113644. doi: 10.1016/j.bbr.2021.113644, PMID: 34757001 PMC8671323

[82] Blackwood CA McCoy MT Ladenheim B Cadet JL. Oxycodone self-administration activates the mitogen-activated protein kinase/mitogen- and stress-activated protein kinase (MAPK-MSK) signaling pathway in the rat dorsal striatum. Sci Rep. (2021) 11:2567. doi: 10.1038/s41598-021-82206-3, PMID: 33510349 PMC7843984

[83] Fan X-Y Shi G He X-J Li X-Y Wan Y-X Jian L-Y. Oxytocin prevents cue-induced reinstatement of oxycodone seeking: involvement of DNA methylation in the hippocampus. Addict Biol. (2021) 26:e13025. doi: 10.1111/adb.13025, PMID: 33609013

[84] Hong Q Xu W Lin Z Liu J Chen W Zhu H. Role of GABRD gene methylation in the nucleus Accumbens in heroin-seeking behavior in rats. Front Pharmacol. (2020) 11:612200. doi: 10.3389/fphar.2020.612200, PMID: 33551813 PMC7859445

[85] Zhang J-J Jiang F-Z Zheng W Duan Y Jin S-B Shen F. DNMT3a in the hippocampal CA1 is crucial in the acquisition of morphine self-administration in rats. Addict Biol. (2020) 25:e12730. doi: 10.1111/adb.12730, PMID: 30950138

[86] Toyama K Kiyosawa N Watanabe K Ishizuka H. Identification of circulating miRNAs differentially regulated by opioid treatment. Int J Mol Sci. (2017) 18:1991. doi: 10.3390/ijms18091991, PMID: 28926935 PMC5618640

[87] Gu W-J Zhang C Zhong Y Luo J Zhang C-Y Zhang C. Altered serum microRNA expression profile in subjects with heroin and methamphetamine use disorder. Biomed Pharmacother. (2020) 125:109918. doi: 10.1016/j.biopha.2020.109918, PMID: 32036213

[88] Sim S-E Lim C-S Kim J-I Seo D Chun H Yu N-K. The brain-enriched MicroRNA miR-9-3p regulates synaptic plasticity and memory. J Neurosci. (2016) 36:8641–52. doi: 10.1523/JNEUROSCI.0630-16.2016, PMID: 27535911 PMC6601897

[89] Zhang Y Wang Y Wang L Bai M Zhang X Zhu X. Dopamine receptor D2 and associated microRNAs are involved in stress susceptibility and resistance to escitalopram treatment. Int J Neuropsychopharmacol. (2015) 18:pyv025. doi: 10.1093/ijnp/pyv025, PMID: 25740916 PMC4571637

[90] Tapocik JD Ceniccola K Mayo CL Schwandt ML Solomon M Wang B-D. MicroRNAs are involved in the development of morphine-induced analgesic tolerance and regulate functionally relevant changes in Serpini1. Front Mol Neurosci. (2016) 9:20. doi: 10.3389/fnmol.2016.00020, PMID: 27047334 PMC4805586

[91] Eipper-Mains JE Kiraly DD Palakodeti D Mains RE Eipper BA Graveley BR. microRNA-Seq reveals cocaine-regulated expression of striatal microRNAs. RNA. (2011) 17:1529–43. doi: 10.1261/rna.2775511 21708909 PMC3153976

[92] Mavrikaki M Anastasiadou E Ozdemir RA Potter D Helmholz C Slack FJ. Overexpression of miR-9 in the nucleus Accumbens increases oxycodone self-administration. Int J Neuropsychopharmacol. (2019) 22:383–93. doi: 10.1093/ijnp/pyz015, PMID: 30989210 PMC6545539

[93] Marie N Canestrelli C Noble F. Role of pharmacokinetic and pharmacodynamic parameters in neuroadaptations induced by drugs of abuse, with a focus on opioids and psychostimulants. Neurosci Biobehav Rev. (2018) 106:217–26. doi: 10.1016/j.neubiorev.2018.06.006, PMID: 30340773

[94] Le Marec T Marie-Claire C Noble F Marie N. Chronic and intermittent morphine treatment differently regulates opioid and dopamine systems: a role in locomotor sensitization. Psychopharmacology. (2011) 216:297–303. doi: 10.1007/s00213-011-2223-6, PMID: 21340469

[95] Allouche S Le Marec T Noble F Marie N. Different patterns of administration modulate propensity of methadone and buprenorphine to promote locomotor sensitization in mice. Prog Neuro-Psychopharmacol Biol Psychiatry. (2013) 40:286–91. doi: 10.1016/j.pnpbp.2012.10.013, PMID: 23103550

[96] Yu S Zhang X Sun Y Peng Y Johnson J Mandrell T. Pharmacokinetics of buprenorphine after intravenous administration in the mouse. J Am Assoc Lab Anim Sci. (2006) 45:12–6., PMID: 16642964

[97] Liu Y Liang J Yan L Su R Wu C Gong Z. Effects of l-tetrahydropalmatine on locomotor sensitization to oxycodone in mice. Acta Pharmacol Sin. (2005) 26:533–8. doi: 10.1111/j.1745-7254.2005.00101.x, PMID: 15842769

[98] Kvello AMS Andersen JM Boix F Mørland J Bogen IL. The role of 6-acetylmorphine in heroin-induced reward and locomotor sensitization in mice. Addict Biol. (2020) 25:e12727. doi: 10.1111/adb.12727, PMID: 30788879

[99] Niikura K Ho A Kreek MJ Zhang Y. Oxycodone-induced conditioned place preference and sensitization of locomotor activity in adolescent and adult mice. Pharmacol Biochem Behav. (2013) 110:112–6. doi: 10.1016/j.pbb.2013.06.010, PMID: 23827650 PMC3805792

[100] Koek W. Effects of repeated exposure to morphine in adolescent and adult male C57BL/6J mice: age-dependent differences in locomotor stimulation, sensitization, and body weight loss. Psychopharmacology. (2014) 231:1517–29. doi: 10.1007/s00213-013-3298-z, PMID: 24096538 PMC3969384

[101] Doherty JM Frantz KJ. Attenuated effects of experimenter-administered heroin in adolescent vs. adult male rats: physical withdrawal and locomotor sensitization. Psychopharmacology. (2013) 225:595–604. doi: 10.1007/s00213-012-2847-1, PMID: 22941050 PMC3547165

[102] Martin-Fardon R Weiss F. Modeling relapse in animals. Curr Top Behav Neurosci. (2013) 13:403–32. doi: 10.1007/7854_2012_202, PMID: 22389178 PMC5137943

[103] Weiss F. Neurobiology of craving, conditioned reward and relapse. Curr Opin Pharmacol. (2005) 5:9–19. doi: 10.1016/j.coph.2004.11.001, PMID: 15661620

[104] Rutten K van der Kam EL De Vry J Tzschentke TM. Critical evaluation of the use of extinction paradigms for the assessment of opioid-induced conditioned place preference in rats. Pharmacology. (2011) 87:286–96. doi: 10.1159/000327680, PMID: 21577043

[105] Tzschentke TM Magalas Z De Vry J. Effects of venlafaxine and desipramine on heroin-induced conditioned place preference in the rat. Addict Biol. (2006) 11:64–71. doi: 10.1111/j.1369-1600.2006.00009.x, PMID: 16759338

[106] Rutten K De Vry J Bruckmann W Tzschentke TM. Effects of the NOP receptor agonist Ro65-6570 on the acquisition of opiate- and psychostimulant-induced conditioned place preference in rats. Eur J Pharmacol. (2010) 645:119–26. doi: 10.1016/j.ejphar.2010.07.036, PMID: 20674566

[107] Murphy NP Ly HT Maidment NT. Intracerebroventricular orphanin FQ/nociceptin suppresses dopamine release in the nucleus accumbens of anaesthetized rats. Neuroscience. (1996) 75:1–4. doi: 10.1016/0306-4522(96)00322-3, PMID: 8923516

[108] Murphy NP Maidment NT. Orphanin FQ/nociceptin modulation of mesolimbic dopamine transmission determined by microdialysis. J Neurochem. (1999) 73:179–86. doi: 10.1046/j.1471-4159.1999.0730179.x, PMID: 10386969

[109] Di Giannuario A Pieretti S. Nociceptin differentially affects morphine-induced dopamine release from the nucleus accumbens and nucleus caudate in rats. Peptides. (2000) 21:1125–30. doi: 10.1016/s0196-9781(00)00250-3, PMID: 10998547

[110] Lutfy K Do T Maidment NT. Orphanin FQ/nociceptin attenuates motor stimulation and changes in nucleus accumbens extracellular dopamine induced by cocaine in rats. Psychopharmacology. (2001) 154:1–7. doi: 10.1007/s002130000609, PMID: 11291998 PMC2288655

[111] Zheng F Grandy DK Johnson SW. Actions of orphanin FQ/nociceptin on rat ventral tegmental area neurons in vitro. Br J Pharmacol. (2002) 136:1065–71. doi: 10.1038/sj.bjp.0704806, PMID: 12145107 PMC1573434

[112] Vazquez-DeRose J Stauber G Khroyan TV Xie XS Zaveri NT Toll L. Retrodialysis of N/OFQ into the nucleus accumbens shell blocks cocaine-induced increases in extracellular dopamine and locomotor activity. Eur J Pharmacol. (2013) 699:200–6. doi: 10.1016/j.ejphar.2012.11.050, PMID: 23219985 PMC3570659

[113] Vander Weele CM Porter-Stransky KA Mabrouk OS Lovic V Singer BF Kennedy RT. Rapid dopamine transmission within the nucleus accumbens: dramatic difference between morphine and oxycodone delivery. Eur J Neurosci. (2014) 40:3041–54. doi: 10.1111/ejn.12709, PMID: 25208732 PMC4358739

[114] Towers EB Tunstall BJ McCracken ML Vendruscolo LF Koob GF. Male and female mice develop escalation of heroin intake and dependence following extended access. Neuropharmacology. (2019) 151:189–94. doi: 10.1016/j.neuropharm.2019.03.019, PMID: 30880124 PMC9345532

[115] Kitamura O Wee S Specio SE Koob GF Pulvirenti L. Escalation of methamphetamine self-administration in rats: a dose-effect function. Psychopharmacology. (2006) 186:48–53. doi: 10.1007/s00213-006-0353-z, PMID: 16552556

[116] Ahmed SH Koob GF. Transition from moderate to excessive drug intake: change in hedonic set point. Science. (1998) 282:298–300. doi: 10.1126/science.282.5387.298, PMID: 9765157

[117] Lenoir M Guillem K Koob GF Ahmed SH. Drug specificity in extended access cocaine and heroin self-administration. Addict Biol. (2012) 17:964–76. doi: 10.1111/j.1369-1600.2011.00385.x, PMID: 21995515

[118] Wade CL Vendruscolo LF Schlosburg JE Hernandez DO Koob GF. Compulsive-like responding for opioid analgesics in rats with extended access. Neuropsychopharmacology. (2015) 40:421–8. doi: 10.1038/npp.2014.188, PMID: 25060491 PMC4443956

[119] Blackwood CA Hoerle R Leary M Schroeder J Job MO McCoy MT. Molecular Adaptations in the Rat Dorsal Striatum and Hippocampus Following Abstinence-Induced Incubation of Drug Seeking After Escalated Oxycodone Self-Administration. Mol Neurobiol (2019) 56:3603–3615. doi: 10.1007/s12035-018-1318-z 30155791 PMC6477015

[120] Guha SK Alonso-Caraballo Y Driscoll GS Babb JA Neal M Constantino NJ. Ranking the contribution of behavioral measures comprising oxycodone self-administration to reinstatement of drug-seeking in male and female rats. Front Behav Neurosci (2022) 16:1035350. doi: 10.3389/fnbeh.2022.1035350 36505730 PMC9731098

[121] Geoffroy H. Canestrelli C. Marie N. Noble F. Comparison of the abuse liability of heroin, morphine and oxycodone in a model of intravenous self-administration in rats. Neuroscience Applied (2023) In press.

[122] Blackwood CA Leary M Salisbury A McCoy MT Cadet JL. Escalated Oxycodone Self-Administration Causes Differential Striatal mRNA Expression of FGFs and IEGs Following Abstinence-Associated Incubation of Oxycodone Craving. Neuroscience (2019) 415:173–183. doi: 10.1016/j.neuroscience.2019.07.030 31351142 PMC7448685

[123] Comer SD Sullivan MA Whittington RA Vosburg SK Kowalczyk WJ. Abuse liability of prescription opioids compared to heroin in morphine-maintained heroin abusers. Neuropsychopharmacology (2008) 33:1179–1191. doi: 10.1038/sj.npp.1301479 17581533 PMC3787689

[124] Remillard D Kaye AD McAnally H. Oxycodone’s Unparalleled Addictive Potential: Is it Time for a Moratorium? Curr Pain Headache Rep (2019) 23:15. doi: 10.1007/s11916-019-0751-7 30820686

[125] Kiyatkin EA. Respiratory depression and brain hypoxia induced by opioid drugs: Morphine, oxycodone, heroin, and fentanyl. Neuropharmacology (2019) 151:219–226. doi: 10.1016/j.neuropharm.2019.02.008 30735692 PMC6500744

